# Combination of PI-RADS score and mRNA urine test—A novel scoring system for improved detection of prostate cancer

**DOI:** 10.1371/journal.pone.0271981

**Published:** 2022-08-12

**Authors:** Olga Katzendorn, Christoph A. J. von Klot, Samy Mahjoub, Pouriya Faraj Tabrizi, Nina N. Harke, Hossein Tezval, Susanne Hellms, Jörg Hennenlotter, Mirza S. Baig, Arnulf Stenzl, Ferdinand Seith, Marcel Lafos, Markus A. Kuczyk, Steffen Rausch, Inga Peters

**Affiliations:** 1 Department of Urology and Urologic Oncology, Hanover Medical School, Hanover, Germany; 2 Institute for Diagnostic and Interventional Radiology, Hanover Medical School, Hanover, Germany; 3 Department of Urology, Eberhard Karls University Tübingen, Tübingen, Germany; 4 Diagnostic and Interventional Radiology, Eberhard Karls University Tübingen, Tübingen, Germany; 5 Institute for Pathology, Hanover Medical School, Hanover, Germany; 6 Department of Urology, Krankenhaus Nordwest, Frankfurt a. Main, Germany; Southern Illinois University School of Medicine, UNITED STATES

## Abstract

Available tests to detect clinically significant prostate cancer frequently lead to overdiagnosis and overtreatment. Our study assessed the feasibility of combining a urinary biomarker-based risk score (SelectMDx^®^) and multiparametric MRI outcomes in order to identify patients with prostate cancer on prostate biopsy with increased accuracy and reliability. Samples of 74 men with suspicion of prostate cancer and available multiparametric MRI were analysed in a prospective cross-sectional study design. First-voided urine for determination of *HOXC6* and *DLX1* mRNA levels was collected after digital rectal examination and prior to MRI/ultrasound fusion-guided prostate biopsy. All multiparametric MRI images were centrally reviewed by two experienced radiologists blinded for urine test results and biopsy outcome. The PI-RADS v2 was used. SelectMDx^®^ score, PI-RADS and Gleason Sore were obtained. Associations between Gleason Score, PI-RADS scores and SelectMDx^®^ were assessed using ANOVA and t-test. Sensitivity and specificity were assessed and evaluated as area-under-the-curve of the receiver operating characteristic. Upon biopsy, 59.5% of patients were diagnosed with prostate cancer, whereby 40.6% had high-grade prostate cancer (GS ≥ 7a). SelectMDx^®^ scores were significantly higher for patients with positive biopsy findings (49.07 ± 25.99% vs. 22.00 ± 26.43%; p < 0.001). SelectMDx^®^ scores increased with higher PI-RADS scores. Combining SelectMDx^®^, history of prior biopsy with benign histology and PI-RADS scores into a novel scoring system led to significant prostate cancer detection rates with tiered detection rate of 39%, 58%, 81% and 100% for Gleason grade group II, III, IV, and V, respectively. The area-under-the-curve for our novel sum score in receiver operating characteristic analysis was 0.84. The synergistic combination of two non-invasive tests into a sum score with increased sensitivity may help avoiding unnecessary biopsies for initial prostate cancer diagnosis. For confirmation, further prospective studies with larger sample sizes and univariate and multivariate regression analyses and decision curve analyses are required.

## Introduction

Globally, prostate cancer (PCa) is the second most prevalent cancer and the fifth leading cause of cancer death in men. Incidences vary across countries, depending on the degree of economic development and associated social and life style factors. In 2018, the estimated age-standardised incidence for Europe ranged between 85.7 in Northern Europe and 42.2 per 100,000 in Eastern Europe [1]. As early detection and treatment of aggressive PCa leads to better outcomes, prostate-specific antigen (PSA) testing along with digital rectal examination (DRE) became the primary standard screening method in clinical practice to identify men with PCa at an early stage [2]. PSA levels of > 4.0 ng/mL are commonly used as a threshold value for prostate biopsy. However, limited sensitivity and specificity of PSA testing to detect high-grade PCa led to overdiagnosis and overtreatment in men whose tumours would have remained clinically insignificant during their lifetime [3]. As an invasive procedure, prostate biopsy is associated with potential adverse events such as rectal bleeding, macroscopic haematuria, haematospermia, fever, infection, and urinary retention [4]. Hence there is a need for more accurate, non-invasive tools for the detection of clinically significant disease. In this context, multiparametric magnetic resonance imaging (mpMRI) has proven to be a valuable addition to PCa diagnostics. mpMRI combines conventional T2-weighted anatomical sequences and functional techniques, such as diffusion-weighted imaging (DWI) and dynamic contrast-enhanced (DCE) for the evaluation of functionally abnormal areas in the prostate [5]. mpMRI has high sensitivity and a high negative predictive value for clinically significant PCa and was shown to be superior to PSA testing alone [6,7]. However, high-grade PCa still remained undetected in 2.3–20% of patients with a negative mpMRI [8].

With the aim to further advance the diagnostic decision-making process, several blood and urine biomarkers have been evaluated over the recent years with regard to their predictive value. SelectMDx^®^ (MDxHealth, Irvine, California, USA) is a clinically validated, commercially available biomarker-based risk score developed to assess the probability of detecting high-grade PCa on prostate biopsy using urinary *HOXC6* and *DLX1* mRNA expression levels combined with traditional clinical risk factors, such as serum PSA, PSA density, DRE status, age, and family history of PCa. The risk score achieved a negative predictive value of 98% for significant PCa [9]. A subsequent observational study showed promising results regarding the correlation between the SelectMDx^®^ score with mpMRI outcomes [8]. Based on these results we examined the feasibility of combining SelectMDx^®^ and mpMRI (Prostate Imaging Reporting and Data System (PI-RADS) scores) with the aim to develop a screening method that can accurately and reliably predict the presence of clinically significant PCa through non-invasive means and thus reduce the number of unnecessary biopsies.

## Materials and methods

### Study design

This prospective cross-sectional observational study analysed a referred sample of men with suspicion of PCa and available mpMRI scan of the prostate. Men with clinical suspicion of PCa who were referred to our centres (Hanover Medical School and University of Tübingen, Germany) for prostate biopsy between February 2018 and February 2020 were eligible to participate in this study. The prospective analysis of anonymous data was approved by the Ethics Committees of Hanover Medical School, Germany (ref. no. 3580–2017) and University of Tübingen, Germany (ref. no. 379/2010 BO2). All subjects gave written informed consent in accordance with the Declaration of Helsinki.

### SelectMDx^*®*^

First-voided urine was collected after DRE, prior to performing prostate biopsies at the same day. On the day of collection, the urine samples were shipped to an external laboratory (MDxHealth B.V., Nijmegen, The Netherlands) for analysis. A detailed description of the assays for measuring expression levels of *HOXC6* and *DLX1* has been previously published [9]. In brief, assays were performed using a prototype amplification kit (Labo Biomedical Products BV, Rijswijk, The Netherlands). RNA was isolated out of 1 mL urine using the MagNA Pure 96 instrument (Roche Life Science, Indianapolis, IN, USA). Subsequently, mRNA levels of *HOXC6* and *DLX1* were determined using one-step reverse transcription quantitative polymerase chain reaction.

### Prostate biopsies

MRI/ultrasound fusion-guided prostate biopsy (median of 16 cores) was performed per hospitals standard procedure and evaluated by an experienced genitourinary pathologist (M.L.). Either transrectal or transperineal approach was performed for MRI fusion-guided prostate biopsy depending on location of the target lesion in MRI. Histological grading was assessed according to the Gleason grading system as well as the Gleason Grade (GG) Groups [10,11].

### Prostate mpMRI

Most patients provided mpMRIs from their referring private practice. All mpMRIs were centrally reviewed by two experienced uro-radiologists (S.H. and F.S.) blinded for the molecular urine test scores and biopsy outcomes. The T2-weighted (T2W) images were used to assess prostate anatomy. Diffusion-weighted imaging (DWI) and dynamic contrast-enhanced (DCE) MRI were used as functional techniques. The PI-RADS version 2 (v2) was used for grading the lesions in all three mpMRI sequences. For each patient a final PI-RADS score from 1 to 5 was determined [12,13].

### Statistical analysis

Statistical analyses were performed using R statistical software [14]. For descriptive data presentation, categorical data was shown with absolute numbers and percentages. Continuous variables were presented with either the mean and the standard deviation or the median with range. For between group comparison, a two-sided t-test and analysis of variance (ANOVA) was applied. For comparison of categorical data, Fisher’s exact test was used. Diagnostic performance of tests in terms of sensitivity and specificity were assessed and evaluated as area-under-the-curve (AUC) of the receiver operating characteristic (ROC). Two-sided p-values of < 0.05 were considered to indicate statistical significance. For patient’s details, we provide the anonymized minimal data set underlying our results described in our manuscript in S1 Table.

## Results

### Study population

In total, data from 74 men were included in the analysis. Patient characteristics, mpMRI, SelectMDx^®^ and biopsy outcomes are summarised in Table 1. Median age was 66.8 years (range 45–80) and mean (SD) PSA level was 8.74 (5.77) ng/ml. Mean (SD) SelectMDx^®^ score was 38.1 (29.2)%. Upon biopsy, 59.5% of patients were diagnosed with PCa, whereby 40.6% had clinically significant PCa (GS ≥ 7a).

**Table 1:** Patient’s characteristics.

Parameter	Total cohort; n = 74
Median age (range), years	66.8 (45–80)
Mean prostate volume, mL	58.0 (41.91)
Mean PSA, ng/mL	8.74 (5.77)
Prior biopsy with benign histology, %	29 (39.2)
mpMRI, n (%)	
PI-RADS 2	16 (21.6)
PI-RADS 3	14 (18.9)
PI-RADS 4	34 (45.9)
PI-RADS 5	10 (13.5)
Mean SelectMDx^®^ score, %	38.1 (29.23)
Mean SelectMDx^®^ score for clinically significant cancer, %	26.1 (18.7)
Biopsy results, n (%)	
no PCa	30 (40.5)
PCa	44 (59.5)
Gleason 6	14 (18.9)
Gleason 7a	19 (25.7)
Gleason 7b	4 (5.4)
Gleason 8	3 (4.1)
Gleason 9	4 (5.4)
Median number of cores upon biopsy, n (range)	16 (2–25)
Positive findings in targeted biopsies, n (%)	30 (40.5)
Positive findings in systematic biopsies, n (%)	36 (48.6)

mpMRI, multiparametric magnetic resonance imaging; PCa, prostate carcinoma; PI-RADS, Prostate Imaging–Reporting and Data System; PSA, prostate-specific antigen.

### Association of SelectMDx^®^ scores with histological characteristics

**Fig 1:**
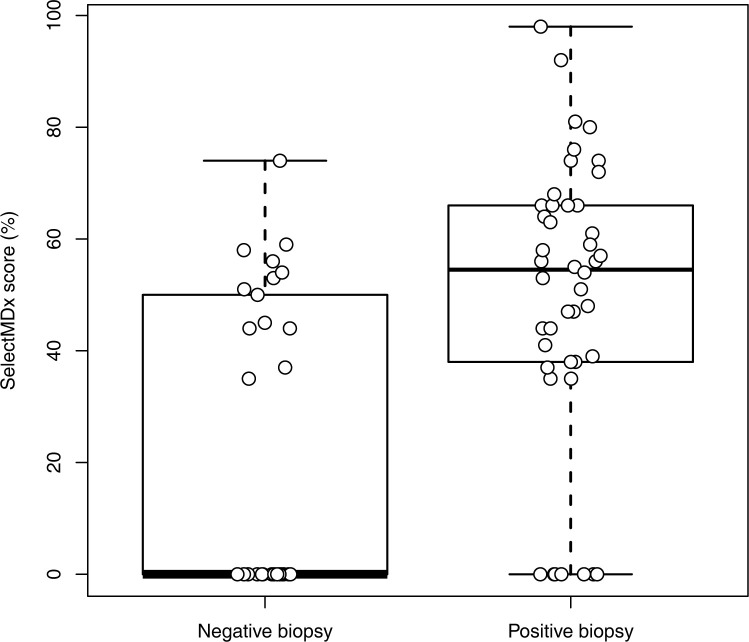
Box plot depicting SelectMDx® scores for negative vs. positive biopsy findings.

**Fig 2:**
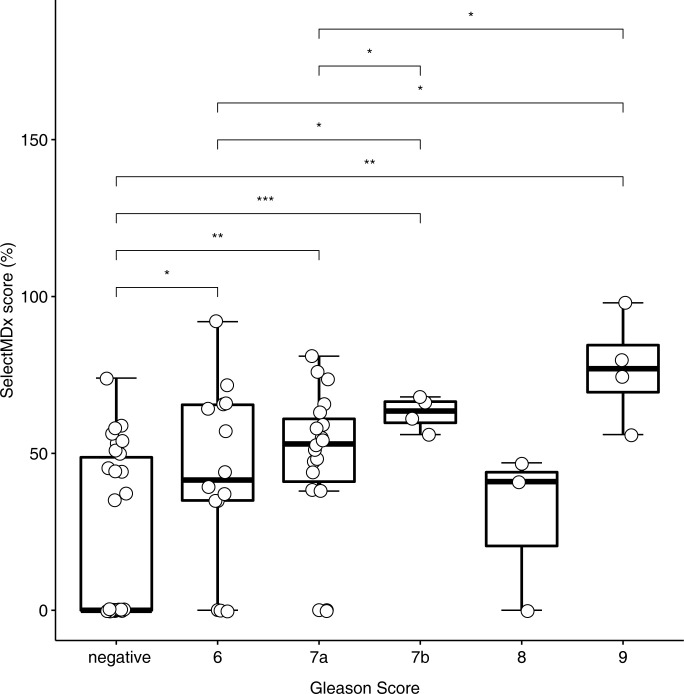
Box plot depicting SelectMDx^®^ scores for different Gleason scores. Each paired boxplot with a statistically significance in means, was indicated using asterisks with the following, standardized significance codes: * p < 0.05, ** p < 0.01, *** p < 0.001. Of note, when dichotomizing into cohorts with patients ≤GS 7a and >7a, a higher GS was associated with a significantly higher SelectMDx® score (58.82 ± 25.08% vs. 34.48 ± 28.55%; p = 0.01).

Differences in the distribution of SelectMDx^®^ scores between positive and negative biopsy findings were analysed using a t-test. SelectMDx^®^ scores were significantly higher for patients with positive biopsy findings (49.07 ± 25.99% vs. 22.00 ± 26.43%; p < 0.001; Fig 1). When dichotomising the cohort into patients ≥ GS 7a (3 + 4), a higher GS was associated with a significantly higher SelectMDx^®^ score (58.82 ± 25.08% vs. 34.48 ± 28.55%; p = 0.01; Fig 2).

### Association of SelectMDx^®^ scores with mpMRI findings

**Fig 3:**
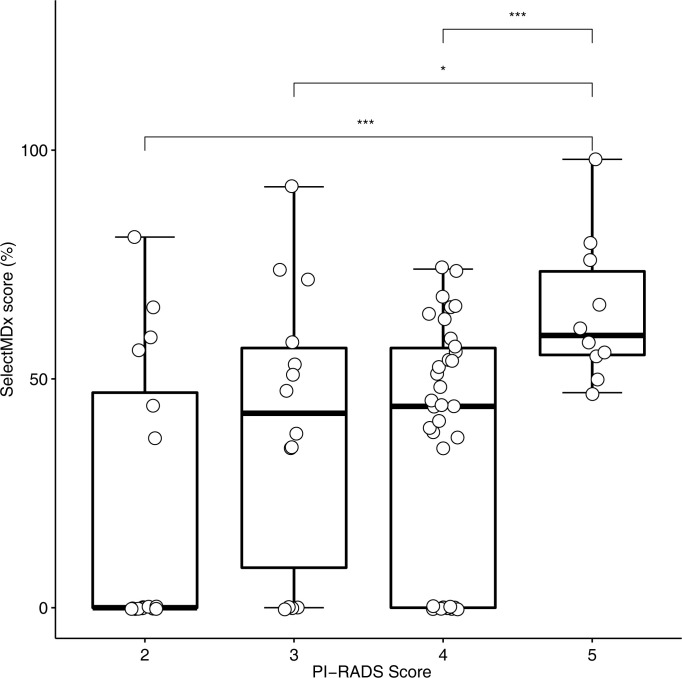
Box plot depicting SelectMDx^®^ scores for different PI-RADS scores. Each paired boxplot with a statistically significance in means, was indicated using asterisks with the following, standardized significance codes: * p ≤ 0.05, ** p ≤ 0.01, *** p ≤ 0.001. The differences in means of SelectMDx^®^ results according to PI-RADS Scores 2–5 were significant in ANOVA (p = 0.002).

The distribution of SelectMDx^®^ scores for PI-RADS scores 2–5 was analysed by means of ANOVA. SelectMDx^®^ scores increased with higher PI-RADS scores. The differences in means of SelectMDx^®^ results according to PI-RADS 2–5 were significant in ANOVA (p = 0.002, Fig 3).

### Association of sensitivity and specificity on detection of PCa for SelectMDx^®^ and PI-RADS

**Fig 4:**
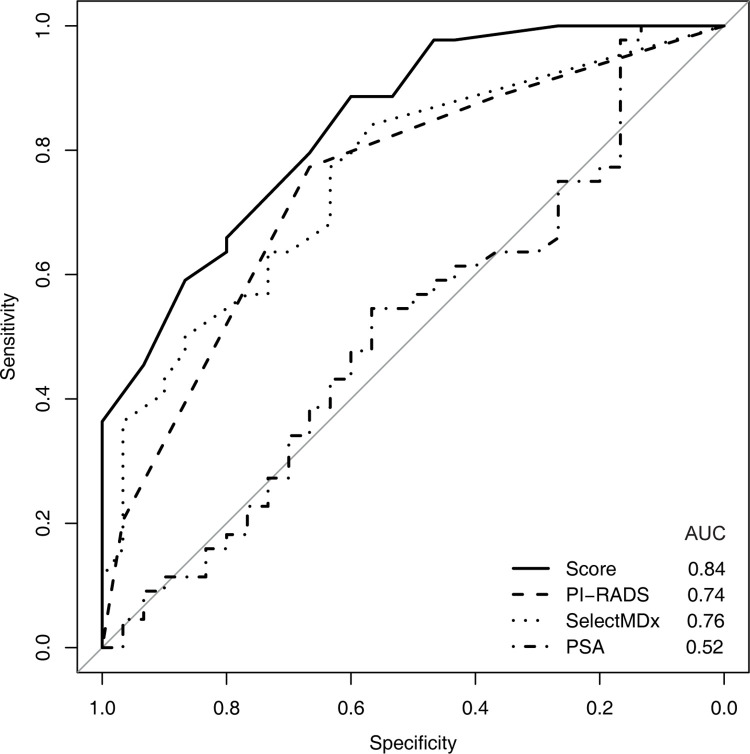
ROC curve for PI-RADS, SelectMDx^®^ urine test marker, PSA and the novel scoring system (combination of PI-RADS, history of prior biopsy and SelectMDx^®^).

The relationship of sensitivity and specificity of SelectMDx^®^ and PI-RADS on the detection of PCa in our cohort was analysed via ROC curve. The AUC for the prediction of PCa for SelectMDx^®^ was 0.76, for PSA 0.52 and for PI-RADS 0.74 (Fig 4).

### Novel scoring system combining SelectMDx^®^ and PI-RADS increased the detection rate of PCa

On the basis of our findings regarding higher SelectMDx^®^ scores for patients with positive biopsies, we developed a novel scoring system combining SelectMDx^®^, PI-RADS scores and information upon prior biopsy. Table 2 summarizes the sum scores annotated to the corresponding GG groups: Patients with and without prior biopsy with benign histology were given 0 or 1 points, respectively. Patients with and without PI-RADS Score of ≥ 4 were given 7 or 0 points and points from SelectMDx^®^ scores (0–100%) were added as the rounded value of 1/10th of the percentage, resulting in 0–10 points for the SelectMDx^®^ score. The detection rate of PCa for 0 (GG I), 1–5 (GG II), 6–10 (GG III), 11–15 (GG IV) and > 15 (GG V) points were 0%, 38.5%, 57.9%, 81.3%, and 100%, respectively. The detection rates for PCa according to our score were statistically significant (p < 0.001) and the AUC for the prediction of PCa was 0.84 (Fig 4).

**Table 2:** Novel sum score derived from the combination of PI-RADS, history of prior biopsy and SelectMDx^®^ score.

PI-RADS / / history of biopsy /	Novel sum Score (% detection rate)										
SelectMDx Score (%)	<5	10	20	30	40	50	60	70	80	90	100
PI-RADS ≤3 + prior negative biopsy	0 (0)	1 (38.5)	2 (38.5)	3 (38.5)	4 (38.5)	5 (38.5)	6 (57.9)	7 (57.9)	8 (57.9)	9 (57.9)	10 (57.9)
PI-RADS ≤3 + no prior biopsy	1 (38.5)	2 (38.5)	3 (38.5)	4 (38.5)	5 (38.5)	6 (57.9)	7 (57.9)	8 (57.9)	9 (57.9)	10 (57.9)	11 (81.3)
PI-RADS ≥4 + prior negative biopsy	7 (57.9)	8 (57.9)	9 (57.9)	10 (57.9)	11 (81.3)	12 (81.3)	13 (81.3)	14 (81.3)	15 (81.3)	16 (100)	17 (100)
PI-RADS ≥4 + no prior biopsy	8 (57.9)	9 (57.9)	10 (57.9)	11 (81.3)	12 (81.3)	13 (81.3)	14 (81.3)	15 (81.3)	16 (100)	17 (100)	18 (100)

Novel sum score ranges from 0–18 points. Prostate cancer risk was assessed with Gleason Grade (GG) Group and was distributed as followed

GG I: 0 points, GG II: 1–5 points, GG III: 6–10 points, GG IV: 11–15 points and GG V: ≥15 points.

Patients with or without prior biopsy with benign histology were given 0 or 1 points, patients with or without PI-RADS Score of ≥ 4 were given 7 or 0 points, afterwards points from SelectMDx® scores (0–100%) were added as the rounded value of 1/10th of the percentage, resulting in 0–10 points for the SelectMDx® score.

The detection rate of PCa for 0 (GG I), 1–5 (GG II), 6–10 (GG III), 11–15 (GG IV) and > 15 (GG V) points were 0%, 38.5%, 57.9%, 81.3%, and 100%, respectively.

## Discussion

In order to improve the quality of PCa diagnostics, we have developed a novel score by combining parameters from two established non-invasive diagnostic tools. Both, mpMRI and SelectMDx^®^ individually cover multiple parameters, which already make them highly specific per se. mpMRI combines anatomical (T2-weighted sequences) and functional techniques (DWI, DCE MRI) for PCa detection and tumour localisation [5]. Yet, reported percentages of 2.3–20% for missed high-grade PCa imply that there is room for improvement [8]. The SelectMDx^®^ score includes clinical parameters like PSA, family history and DRE in addition to mRNA expression levels of genes *HOXC6* and *DLX1*. Regulating genes with both oncogenic and tumour suppressor activities, as well as several genes important for prostate morphogenesis and metastasis to the bone, *HOXC6* is frequently overexpressed in patients with PCa [15]. *DLX1* is involved in neuroendocrine-epithelial differentiation, which is characteristic of aggressive PCa [16]. Both the *HOXC6* and *DLX1* biomarkers have been shown to have independent value in predicting Gleason ≥7 PCa on biopsy [17]. mRNA assays for SelectMDx^®^ can be performed on whole urine samples, which precludes that mRNA yield is compromised by labour-intensive and time-consuming urine-processing procedures [18]. As the only available test based on mRNA expression levels, the SelectMDx^®^ test predicts high-grade PCa with 98% negative predictive value, still leaving room for improvement [9].

Recent reviews on available biomarkers concluded that novel combinations of newer biomarkers or mpMRI with existing risk-predicting models may have the potential of improving the accuracy of screening tools [19]. To this end, a previous observational study investigated the association between SelectMDx^®^ score, mpMRI outcomes, and biopsy GS [8]. The authors found a positive association between the SelectMDx^®^ score and the final PI-RADS score, as well as a statistically significant difference in the SelectMDx^®^ score between PI-RADS 3 and 4 (p < 0.01) and between PI-RADS 4 and 5 (p < 0.01) [8]. However, these results were limited by the retrospective study design and preselection of patients, who had undergone a SelectMDx^®^ urine test and prostate biopsies in previous study protocols [9]. We chose a prospective approach by including patients with suspicion of PCa who were referred to our centres for prostate biopsy. Furthermore, generating a sum score from the stratified SelectMDx^®^ (<5%,10%,20% … 100%), history of prior biopsy and PI-RADS (≤ 3, ≥ 4) scores led to statistically significant detection rates with tiered detection rates of 39%, 58%, 81%, and 100% for the sum scores of 1–5 (GG II), 6–10 (GG III), 11–15 (GG IV) and > 15 (GG V) points, respectively.

Combining SelectMDx^®^, history of prior biopsy and PI-RADS score into a novel scoring system has proven to be feasible and potentially improves the prediction of PCa in our pilot cohort of 74 men. Further prospective studies with larger sample sizes as well as univariate and multivariate regression analyses and decision curve analyses are required to confirm our results. Cost-effectiveness analyses conducted in several European countries indicated that healthcare costs can be saved when the commercially available SelectMDx^®^ is applied in the initial diagnosis of PCa. The significant presence of overtreatment in the current standard of care was the driving factor that resulted in the beneficial outcomes with SelectMDx^®^ [20,21]. The synergistic combination of two non-invasive tests into a sum score with increased sensitivity compared to the individual scores alone may further reduce the number of unnecessary biopsies for initial PCa diagnosis and prevent the detection of clinically insignificant PCa. Biopsies are not only uncomfortable for the patient, but they are also associated with adverse events inherent to invasive procedures. Avoiding unnecessary biopsies would therefore spare the patient physical discomfort in addition to the psychological burden associated with the diagnosis of clinically insignificant PCa. Besides being potentially helpful in the decision-making process, the novel scoring system could also support the selection of patients with low-risk PCa for active surveillance. However, the prognostic role of the combined SelectMDx^®^/PI-RADS test requires additional long-term studies. Our results strongly support the effort of an ongoing national multicenter trial conducted by the University of Cologne (DRKS-ID: DRKS00019892) to further evaluate this issue.

This study has few limitations. Data were obtained from two centres only, which might cause selection bias. However, bias was minimised by including patients consecutively as they presented at our centres with suspicion of PCa. The lack of centrally performed MRIs, usually a major limitation, is offset by a central review of all MRIs by two experienced uro-radiologists who were blinded for the urine test scores and biopsy outcomes. DRE as part of the multiparametric SelectMDx^®^ score is subject to interobserver variability. Nevertheless, the SelectMDx^®^ score has been shown to enable objective clinical risk assessment and patient management by remaining the strongest, most significant predictor of patient risk compared with other clinically relevant risk assessment algorithms, such as prostate cancer antigen 3 and the Prostate Cancer Prevention Trial risk calculator [9].

## Conclusions

Combining SelectMDx^®^, history of prior biopsy and PI-RADS into a novel scoring system led to statistically significant PCa detection rates with tiered detection rates of 39%, 58%, 81%, and 100%. The synergistic combination of two non-invasive tests into a sum score with increased sensitivity compared to the individual scores alone has the potential to avoid unnecessary biopsies for initial PCa diagnosis and prevent the detection of clinically insignificant PCa. Before this novel score can be implemented into clinical practice, our results need to be confirmed in further prospective studies with larger sample sizes as well as univariate and multivariate regression analyses and decision curve analysis.

## Supporting information

S1 Table Anonymized patient’s raw data. (PDF)
