# Supplementary material for: Combination of PI-RADS score and mRNA urine test—A novel scoring system for improved detection of prostate cancer
Source: PLoS One. 2022 Aug 12;17(8):e0271981. doi: 10.1371/journal.pone.0271981 (PMC9374213; doi:10.1371/journal.pone.0271981)
Supplement: S1 Table — (PDF) [file pone.0271981.s001.pdf]

Patient ID	Age	Prostate volume (ml)	PSA (ng/ml)	Prior biopsy (0=no, 1=yes)	PIRADS	SelectMDX score_all_pca (%)	SelectMDX sore_clinical significant pca (%)	Gleason Score in biopsy
1	49	35	5,92	1	2	0	0	0
2	48	35	6,63	0	4	35	11	6
3	79	122	12	1	4	0	0	8
4	71	88	14,7	1	4	38	13	7a
5	80	NA	8,09	1	5	50	23	0
6	71	70	4,15	1	4	0	0	6
7	45	27	3,06	0	3	35	11	6
8	80	40	13,49	1	5	55	27	7a
9	58	110	9,5	0	4	0	0	6
10	72	60	5,84	0	2	0	0	0
11	79	130	9,75	1	2	0	0	0
12	72	54	8,5	0	4	39	14	6
13	74	43	9,8	1	4	44	18	6
14	57	56	6,59	1	3	0	0	0
15	74	23	7,2	1	4	37	12	0
16	57	26	11,4	1	3	47	20	7a
17	60	40	7,68	1	4	0	0	7a
18	70	25	2,15	0	5	47	20	8
19	48	28	6,84	1	3	35	11	0
20	76	184	25	0	5	66	38	7b
21	71	33	13,34	1	4	74	48	9
22	74	49	9,57	0	4	66	38	7a
23	65	180	16,48	1	2	0	0	0
24	48	46	3,85	0	4	0	0	6
25	61	17	9,24	0	3	92	73	6
26	78	47	9,64	1	4	74	48	7a
27	52	33	4,82	0	3	0	0	0
28	74	94	20,4	0	5	80	55	9
29	67	62	4,77	1	3	0	0	0
30	77	98	21,8	1	5	76	50	7a
31	49	34	3,51	0	4	0	0	7a
32	70	78	9,11	0	4	44	17	7a
33	77	45	1,57	1	3	53	25	0
34	60	110	9,29	0	4	45	18	0
35	65	54	8,16	0	4	0	0	0
36	72	107	28,1	0	2	56	28	0
37	70	40	8,75	0	3	51	23	0
38	76	45	5,84	0	5	61	33	7b
39	51	29	1,09	0	4	0	0	0
40	69	37	2,33	0	2	0	0	0
41	62,679	57,5	10,5	0	4	48	21	7a
42	65,962	37	4	0	4	51	23	7a
43	71,945	50	3,88	0	2	66	38	6
44	66,685	38	2,92	1	4	53	25	7a
45	68,51	60	10,36	1	4	59	31	0
46	69,474	54,7	8,25	1	4	54	26	7a
47	77,107	16	3,4	1	2	81	56	7a
48	61,611	67	8,49	1	2	0	0	0
49	67,896	21,94	5,85	1	3	58	30	0
50	57,589	57	9,53	0	2	0	0	0
51	56,178	60	13	0	2	44	17	0
52	54,104	35	6	1	2	0	0	0
53	58,8	36,3	11,5	0	3	74	48	0
54	63,589	37,5	8,4	0	4	66	38	6
55	73,485	56	6	0	2	59	31	7a
56	57,025	132	14,2	1	4	44	17	0
57	65,225	39,3	9,4	0	4	68	40	7b
58	72,416	57	5,26	0	5	56	28	9
59	59,238	31	8,3	0	4	56	28	7b
60	68,019	27,51	0,77	0	3	0	0	0
61	63,488	56	9,63	0	5	58	30	7a
62	77,129	23,3	25	0	5	98	93	9
63	70,581	30	5,35	0	4	64	36	6
64	70,912	50	7	0	4	63	35	7a
65	64,929	40	3,45	0	4	41	15	8
66	71,836	40	0,99	0	4	0	0	0
67	60,866	37	5,66	0	2	37	12	6
68	66,699	65	13,7	1	2	0	0	0
69	65,934	266	23,8	0	4	54	26	0
70	62,693	35	10	0	4	57	29	6
71	66,258	67	4,37	0	2	0	0	7a
72	56,849	37	4,29	1	4	0	0	0
73	60,219	48	5,08	0	3	38	13	7a
74	78,033	32	8,6	0	3	72	54	6
